# De-Regulated MicroRNAs in Pediatric Cancer Stem Cells Target Pathways Involved in Cell Proliferation, Cell Cycle and Development

**DOI:** 10.1371/journal.pone.0061622

**Published:** 2013-04-17

**Authors:** Patricia C. Sanchez-Diaz, Tzu-Hung Hsiao, Judy C. Chang, Dong Yue, Mimi C. Tan, Hung-I Harry Chen, Gail E. Tomlinson, Yufei Huang, Yidong Chen, Jaclyn Y. Hung

**Affiliations:** 1 Greehey Children’s Cancer Research Institute, University of Texas Health Science Center San Antonio, San Antonio, Texas, United States of America; 2 Division of Hematology and Oncology, Department of Pediatrics, University of Texas Health Science Center San Antonio, San Antonio, Texas, United States of America; 3 Department of Epidemiology and Biostatistics, University of Texas Health Science Center San Antonio, San Antonio, Texas, United States of America; 4 Department of Electrical and Computer Engineering, University of Texas at San Antonio, San Antonio, Texas, United States of America; 5 Cancer Therapy and Research Center, University of Texas Health Science Center San Antonio, San Antonio, Texas, United States of America; University of Barcelona, Spain

## Abstract

**Background:**

microRNAs (miRNAs) have been implicated in the control of many biological processes and their deregulation has been associated with many cancers. In recent years, the cancer stem cell (CSC) concept has been applied to many cancers including pediatric. We hypothesized that a common signature of deregulated miRNAs in the CSCs fraction may explain the disrupted signaling pathways in CSCs.

**Methodology/Results:**

Using a high throughput qPCR approach we identified 26 CSC associated differentially expressed miRNAs (DEmiRs). Using BCmicrO algorithm 865 potential CSC associated DEmiR targets were obtained. These potential targets were subjected to KEGG, Biocarta and Gene Ontology pathway and biological processes analysis. Four annotated pathways were enriched: cell cycle, cell proliferation, p53 and TGF-beta/BMP. Knocking down *hsa-miR-21-5p*, *hsa-miR-181c-5p* and *hsa-miR-135b-5p* using antisense oligonucleotides and small interfering RNA in cell lines led to the depletion of the CSC fraction and impairment of sphere formation (CSC surrogate assays).

**Conclusion:**

Our findings indicated that CSC associated DEmiRs and the putative pathways they regulate may have potential therapeutic applications in pediatric cancers.

## Introduction

MicroRNAs (miRNA) are an abundant class of small (∼22 nucleotides) non-coding single strand RNAs (ncRNA) that regulate gene expression at a post-transcriptional level. These regulatory ncRNAs play important roles in the control of many biological processes and their deregulation has been implicated in a variety of pathological conditions, including many cancers.

Emerging evidence supports the notion that most cancers have their own “stem cells”, referred to as “cancer stem cells” (CSCs). This reservoir of stem-like cells within the tumor mass has the ability to self-renew and to sustain the relentless growth of the mass. Several studies have shown that miRNAs are involved in the self-renewal and cell-fate decisions of stem cells, control of cell cycle and maintaining the balance of cell proliferation, differentiation and apoptosis [1],[2],[3],[4]. Recently, studies have shown that mechanisms regulating the self-renewal nature of these cells are dysfunctional in cancer stem cells [5]. We propose that the defining features of CSCs can be described in terms of a coherent pattern of gene expression that is regulated by specific, aberrantly expressed miRNAs coordinated towards the maintenance and self-renewal of cancer stem cells. The regulation of self-renewal and the ability to generate progeny is based on common genes and mechanisms. This subset of genes whose expression is deregulated by the miRNAs may explain the disrupted signaling pathways of the CSCs.

In conventional gene expression data analysis, enrichment analysis of differential expressed genes has been successfully utilized to explore the underlying signaling pathways and gene ontology functions [6],[7],[8]. The strategy provides an interpretation process from differential expressed genes to pathways or functions via a gene set enrichment algorithms [9]. Recently, several algorithms have been developed to predict miRNA target genes through sequence homology between 3′ UTR and miRNA seed region [10],[11],[12]. Through the predicted target genes of miRNAs [11], several studies explored the methods to annotate functions of miRNAs, such as method of pathway targets in conservation enrichment or co-targeting pairs such that the dominant functions might emerge via “hub” miRNAs [13], a permutation-based statistical method that tests for over- or under-representation of miRNA targets in a designated set of target genes [14],[15],[16],[17] and recently released mirDIP that combined 12 target prediction algorithms to form miRNA interaction networks [18]. However, all these algorithms utilize only the target prediction information, regardless of the expression levels of miRNAs, combined with the fact that one miRNA targets over 100 genes [19], making inference to functions or pathways from a set of miRNAs not specific to a biological objective overly complicated.

Different from aforementioned algorithms, we proposed a novel algorithm that combined a set of miRNA target prediction algorithms with a Bayesian scheme to integrate the prediction strength, and then build the miRNA-pathway pair by further integrating the concept of gene set enrichment with a differentially expressed miRNAs (DEmiRs). The enrichment step is achieved by first mapping miRNAs’ expression onto their target genes factored by their prediction strength, then both enrichment score (ES) and the Fisher’s exact test were performed over a gene set or a pathway to explore the putative regulation exerted from DEmiRs.

CSCs have been described in many childhood solid tumors. These tumors are considered as a group of heterogeneous tumors, but with similar and unique genetic features. Recent evidence suggests that the perturbation of the normal development processes is a major factor in childhood solid tumor pathogenesis. The biology of most children cancers differs from adult cancers, for the most part, due to their blastoma origin. Children with congenital genetics disorders frequently develop multiple types of childhood solid tumors. These observations suggest that there might be certain pathways that are shared in a variety of different childhood solid tumors [20].

In this study, we identified common CSCs associated DEmiRs in 6 pediatric solid tumor cell lines. We used a high throughput qRT-PCR approach to survey global miRNA expression in their CSC fraction. We then applied our novel miRNA-pathway association algorithm to elucidate the characteristics of the CSC. In addition, we studied the potential effects of some of the DEmiRs in CSCs by gene silencing.

## Results

### Hierarchical Clustering of the Cancer Stem Cells miRNAs

**Figure 1:**
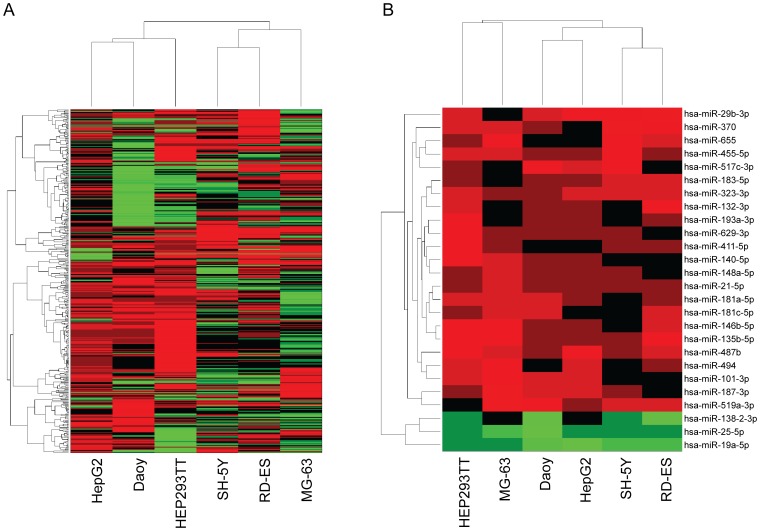
Heatmap of miRNA profile in 6 cell lines. (A). The expression values of 380 DEmiRs were measured and hierarchical clustering was performed. The expression profile of HepG2 and Hep293TT were clustered with Daoy. RD-ES and MG-63 are grouped together with SH-SY5Y. Differentially expressed miRNAs (B). Twenty-six miRNAs with at least 2-fold change in 4 or more cell-lines were selected. Twenty-three miRNAs were up regulated and 3 were down regulated.

Using the neurosphere and ALDEFLUOR® surrogate assays as read-out strategies aimed at enriching a stem-like cancer cell (CSC) population, we had uncovered specific DEmiRs that were dysregulated in this population. We profiled six pediatric cell lines, Hep293TT, HepG2, MG-63, Daoy, SH-SY5Y, and RD-ES using TaqMan MicroRNA Micro Fluid Card Assays and real-time PCR analysis (Applied Biosystems). We performed quality controls examining the scatter plots of all the miRNA expression levels (ΔCt) in the 6 cell lines tested (Figure S1 in File S1). The scatter plots of the ΔCt comparing the stem-like enriched fraction vs. their non stem-like counterparts exhibit concordance (correlation coefficient >0.75). As described in the materials and methods section, we obtained 380 DEmiRs with expression levels 2.2-fold up or down regulated in the CSC fractions compared to their non-cancer stem cell reference fraction. With these 380 DEmiRs, we selected the miRNAs that were differentially expressed in at least 4 out of our 6 cell lines to allow some level of fault-tolerance. The possibility of selecting a DEmiR by random chance was 0.017 (binomial distribution). Hierarchical clustering was performed on these 380 DEmiRs, as shown in Figure 1A. The miRNAs of HepG2 and Hep293TT (hepatoblastoma) and Daoy (medulloblastoma) were clustered together, while RD-ES (Ewing’s sarcoma) and MG-63 (osteosarcoma) and SH-SY5Y (neuroblastoma) were grouped together. A group of 26 DEmiRs (23 up-regulated and 3 down-regulated) were found (Figure 1B), including *hsa-miR-21-5p*, *hsa-miR-148a-5p*, *hsa-miR-181a-5p*, *hsa-miR-323-3p*, and *hsa-miR-487b-3p*, as highly expressed, and *hsa-miR-19a-5p* and *hsa-miR-25-5p*, as down-regulated DEmiRs.

### miRNA Target Prediction

To identify the putative target genes of the DEmiRs, the target gene prediction algorithm, BCmicrO [21] that is described in the Materials and Methods section, was applied to the DEmiRs. In total, 865 putative microRNA target genes (630 up regulated and 235 down regulated) were identified (Figure S2 in File S1). In our analysis each DEmiR on average had 38 predicted target genes, with the exception of *hsa-miR-138-2-3p*, *hsa-miR-655*, and *hsa-miR-101-3p*, each with more than 100 predicted target genes. In addition, we also found that two DEmiRs, *hsa-miR-487b-3p* and *hsa-miR-517c-3p*, had 7 and 25 target genes, respectively, predicted by TargetScan algorithm. However, these DEmiRs did not pass the selection criterion of BCmicrO (see Materials and Methods section).

### Enrichment Analyses of miRNA Regulated Pathways

**Figure 2:**
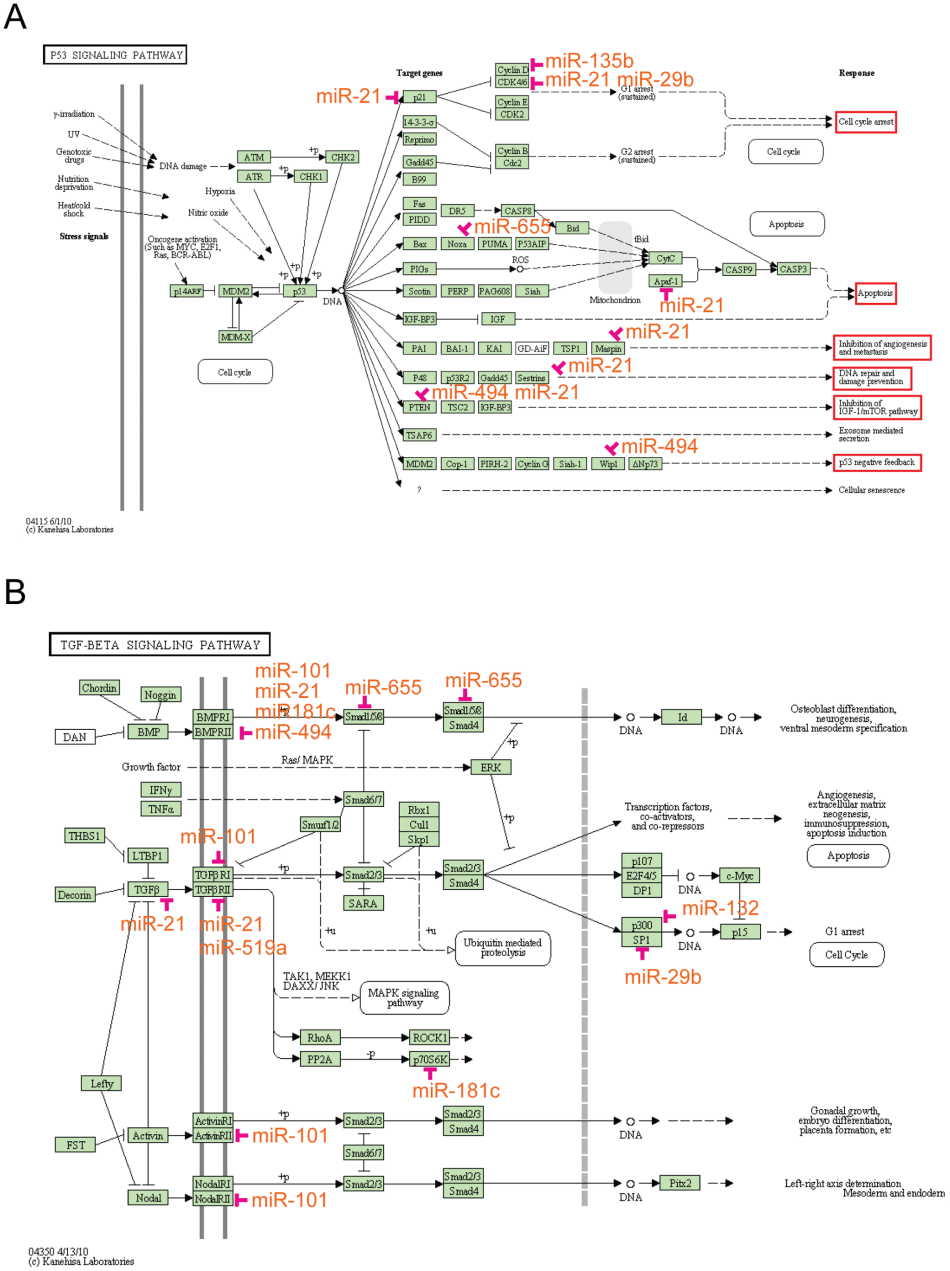
p53 signal pathway of KEGG. (A). The p53 pathway that includes 8 signaling components as defined by KEGG was highly regulated by the CSC associated DEmiRs. Five of the 26 DEmiRs were implicated in this pathway, *hsa-miR-21-5p, hsa-miR-135b, hsa-miR-29, hsa-miR-655, hsa-miR-494*. TGF-beta pathway of KEGG (B). The TGF-beta pathway was also regulated by the CSC associated DEmiRs. Receptor *TGFBR1* was regulated by *hsa-miR-101-3p*, *and* receptor *TGFBR2* was regulated by *hsa-miR-21-5p*, and *hsa-miR-519a-3p*. The receptor *BMPR2* was regulated by *hsa-miR-101-3p, hsa-miR-21-5p, hsa-miR-181c-5p, and hsa-miR-494*. Nodal type II receptor kinase and receptor *ACVR2B* were also regulated by *hsa-miR-101-3p*.

To predict which pathways our 26 CSCs DEmiRs might be regulating we used the miRNA-mRNA pairs obtained as described above for pathway and function enrichment analyses. The gene sets from KEGG, Biocarta pathways and the Gene Ontology (GO) terms of Biological Process were used. We found 8, 12, and 34 items with statistical significance in KEGG pathway, Biocarta pathway and Gene Ontology, respectively (Tables 1, 2, 3). Six out of the 8 enriched items in the KEGG pathway belonged to different cancer types: small cell lung cancer, prostate cancer, chronic myeloid leukemia, glioma, melanoma, and pancreatic cancer. Among the gene sets, many cell cycle and cell proliferation related genes such as *CDKN1A*, *CDKN1B*, *E2F1*, *PTEN* and *RB1* were regulated by the differentially expressed miRNAs (Table S1 in File S1), indicating cell proliferation to be an important function regulated by the CSC associated DEmiRs. The p53 pathway was also enriched since 15% of the genes in this pathway (*P*<0.001, *ES* = 2.18) were targets of our DEmiRs (Table 1). Our method predicted a set of 10 genes implicated in the p53 pathway as potential targets for *hsa-miR-21-5p*, *hsa-miR-29b*, *hsa-miR-135b-5p*, *hsa-miR-494*, and *hsa-miR-655* (Figure 2A). Specific functions of p53 pathway that might be affected by these DEmiRs were cell cycle arrest, apoptosis, inhibition of angiogenesis and metastasis, DNA repair and damage prevention, inhibition of TGF-1/mTor pathway, and p53 negative feedback (Figure 2A, shown boxed out in red).

**Table 1:** CSC associated DEmiRs regulated KEGG pathways.

Pathways (# of genes in pathway)	Number of mirRNAs target genes (%)	Enrichment Score	*P* value
Small Cell Lung Cancer (83)	12 (14%)	2.31	<0.001
P53 Signaling Pathway (66)	10 (15%)	2.18	<0.001
Prostate Cancer (86)	11 (13%)	1.85	<0.001
Chronic Myeloid Leukemia (71)	10 (14%)	2.33	<0.001
Glioma (63)	9 (14%)	2.26	<0.001
Melanoma (67)	8 (12%)	2.43	<0.001
TGF-Beta Signaling Pathway (85)	9 (11%)	2.67	<0.001
Pancreatic Cancer (69)	7 (10%)	2.69	0.001

Significantly regulated KEGG pathways by DEmiRs. Total number of genes in a given pathway or a gene set is provided within the parentheses immediately after the pathway/gene set name. The numbers of genes that are targeted by the DEmiRs are listed in the second column. Enrichment Scores (ESs) were calculated using Eq. 3, and *P* values were evaluated by Fisher’s exact test. Pathways with *P* value <0.5 and ES>1.5 were listed in the table.

**Table 2:** CSC associated DEmiRs regulated BioCarta pathways.

Pathways (# of genes in pathway)	Number of miRNAs target genes (%)	Enrichment Score	*P* value
CTCF Pathway (22)	8 (36%)	2.43	<0.001
Cell Cycle Pathway (22)	6 (27%)	2.23	<0.001
P53 Pathway (16)	5 (31%)	1.75	<0.001
G1 Pathway (27)	6 (22%)	2.20	<0.001
TOB1 Pathway (19)	5 (26%)	2.25	<0.001
ALK Pathway (37)	6 (16%)	2.98	<0.001
RACCYCD Pathway (26)	5 (19%)	2.29	<0.001
TGFB Pathway (18)	4 (22%)	2.28	0.001
PTEN Pathway (18)	4 (22%)	2.09	0.001
TFF Pathway (21)	4 (19%)	1.55	0.001
HIV-I NEF Pathway (56)	6 (11%)	1.73	0.003
Cytokines and Inflammatory Response Pathway (29)	4 (14%)	1.65	0.005

Statistical significance was determined using the same criterion as described in Table 1.

**Table 3:** CSC associated DEmiRs regulated Biological Processes (Gene Ontology Terms).

Gene Ontology: Biological Process Terms (# of genes in pathway)	Number of mirRtarget genes (%)	Enrichment Score	*P* value
Positive Regulation of Bone Mineralization (25)	4 (16.0%)	2.99	0.003
Peptidyl-Threonine Phosphorylation (20)	4 (20.0%)	2.77	0.001
Positive Regulation of Mesenchymal Cell Proliferation (32)	4 (12.5%)	2.66	0.007
Vasculogenesis (54)	6 (11.1%)	2.66	0.002
Blood Vessel Development (56)	8 (14.3%)	2.62	<0.001
BMP Signaling Pathway (51)	6 (11.8%)	2.51	0.001
Negative Regulation of Protein Phosphorylation (26)	4 (15.4%)	2.48	0.003
Negative Regulation of Epithelial Cell Proliferation (40)	8 (20.0%)	2.44	<0.001
Regulation of Gene Expression (66)	7 (10.6%)	2.33	0.001
G1 Phase of Mitotic Cell Cycle (26)	5 (19.2%)	2.29	<0.001
Collagen Fibril Organization (32)	4 (12.5%)	2.26	0.007
Cellular Response to Amino Acid Stimulus (31)	6 (19.4%)	2.23	<0.001
TGF-TGF-Beta Receptor Signaling Pathway (67)	10 (14.9%)	2.19	<0.001
Palate Development (48)	7 (14.6%)	2.16	<0.001
Blastocyst Development (19)	4 (21.1%)	2.08	0.001
Response To Radiation (29)	4 (13.8%)	2.06	0.005
Positive Regulation Of Fibroblast Proliferation (33)	6 (18.2%)	2.04	<0.001
Cellular Response To Hypoxia (30)	5 (16.7%)	2.00	0.001
Skeletal System Morphogenesis (34)	7 (20.6%)	2.00	<0.001
Digestive Tract Development (19)	5 (26.3%)	1.83	<0.001
Wound Healing (67)	8 (11.9%)	1.79	<0.001
Response To Vitamin D (19)	4 (21.1%)	1.74	0.001
Induction Of Apoptosis By Intracellular Signals (43)	6 (14.0%)	1.72	0.001
Response To Organic Substance (90)	9 (10.0%)	1.71	<0.001
Positive Regulation Of B Cell Proliferation (33)	5 (15.2%)	1.70	0.001
Response To Inorganic Substance (24)	5 (20.8%)	1.70	<0.001
Embryo Development (124)	13 (10.5%)	1.69	<0.001
Inner Ear Development (30)	6 (20.0%)	1.69	<0.001
B Cell Differentiation (39)	5 (12.8%)	1.63	0.002
Positive Regulation of SMC Proliferation (36)	6 (16.7%)	1.61	<0.001
Embryo Implantation (31)	5 (16.1%)	1.58	0.001
Cell Growth (45)	5 (11.1%)	1.53	0.004
Chromatin Remodeling (50)	5 (10.0%)	1.52	0.007
Positive Regulation Of PKB Signaling Cascade (40)	5 (12.5%)	1.51	0.003

The selection criterion to determine statistical significance was the same as in Table 1.

Another important pathway in cell-cycle regulation, TGF-beta/BMP pathway, was also enriched (*P = *0.001, *ES* = 2.28; Table 1). Nine genes including 4 receptors, *TGFBR1*, *TGFBR2*, *ACVR2B* and *BMPR2* and one ligand, TGF-beta, were regulated by 8 (*hsa-miR-181c-5p*, *hsa-miR-21-5p, hsa-miR-101-3p*, *hsa-miR-494, hsa-miR-655, hsa-mi-519a-3p, hsa-miR-29b-3p, hsa-miR-132-3p)* of the 26 CSC associated DEmiRs, as shown in Figure 2B. As suggested in Figure 2B, alteration in TGF-beta and BMP receptors might interfere with Smad-dependent signaling in CSCs, perhaps resulting in perturbed growth-arrest, apoptotic and differentiation programs. Thus, the loss of TGF-beta sensitivity causing deregulation of cyclins, CDKs and CDK inhibitors, compounded with dysregulation in p53 pathways supported our prediction that these CSC associated DEmiRs might have a role in the regulation of cell cycle and cell proliferation. The enrichment analysis of Biocarta pathways, further supported the results obtained from the KEGG pathways (Table 1). Four annotated Biocarta pathways, CELLCYCLE, G1, P53, and RACCYCD, were also associated to cell cycle (Table 2, *P*<0.001 for all 4 pathways). Pathways illustrations along with CSC associated DEmiRs target genes are provided in Figure S3 A–D in File S1. The TGF-beta/BMP pathway also showed enrichment in Biocarta analysis (Table 2, *P* = 0.001), therefore confirming our KEGG pathway prediction. Additional pathways obtained in the Biocarta enrichment analysis were CTCF, TOB1, ALK and PTEN pathways (Table 2, *P* value less or equal to 0.001). Some known genes in these pathways are *TGFBR1*, *TGFBR2*, and *TGFB*, *SMAD* family and *PTEN*, further supporting the involvement of our CSC associated DEmiRs in cell proliferation and cell cycle. The biological processes in Gene Ontology were also applied in our enrichment analysis. Thirty-four GO terms were enriched (*P*<0.01, Table 3). Biological processes related to cell proliferation such as Positive Regulation of Mesenchymal Cell Proliferation (*P*<0.007, *ES* = 2.66), Negative Regulation of Epithelial Cell Proliferation (*P*<0.001, *ES* = 2.44), G1 Phase of Mitotic Cell Cycle (*P*<0.001, *ES* = 2.29), Positive Regulation of Fibroblast Proliferation (*P*<0.001, *ES* = 2.04), Positive Regulation of B Cell Proliferation (*P* = 0.001, *ES* = 1.70), Positive Regulation of Smooth Muscle Cell Proliferation (*P*<0.001, *ES* = 1.61) and cell growth (*P* = 0.004, *ES* = 1.53) were enriched in our CSC associated DEmiRs. Some biological processes correlated with cancer biology, vasculogenesis, cellular response to hypoxia, wound healing, and response to radiation, were enriched as well (Table 3). BMP signaling and TGF-beta also showed up as enriched pathways in the GO analysis, similar to KEGG and Biocarta pathway analyses. Signaling pathways such as, positive regulation of protein kinase B signaling cascade, activation of protein kinase C activity by G-protein coupled receptor protein signaling pathway, also showed significant enrichment in the GO analysis (Table 3).

### Functional Evaluation of hsa-miR-21-5p, hsa-miR-181c-5p and hsa-miR-135b-5p In vitro

**Figure 3:**
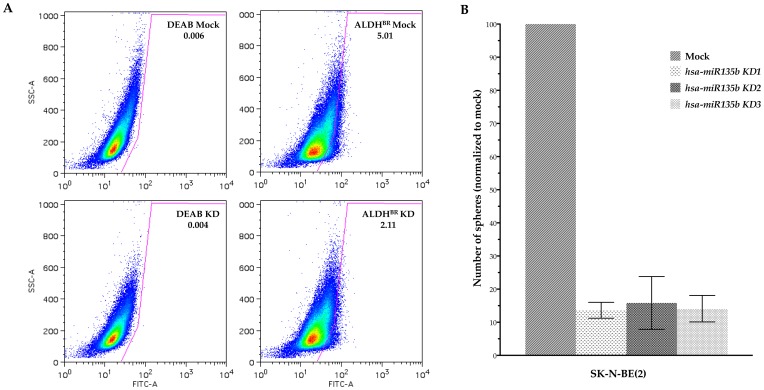
*hsa-miR-21-5p* knockdown on the ALDH^BR^ fraction in Daoy. (A). Approximately a two-fold reduction in the fraction enriched for stem-like cells was observed in the *hsa-miR-21-5p* knockdown. Data shown is representative of three independent experiments. *hsa-miR-135b-5p* inhibition on the ability to form neurospheres in SK-N-NE(2) (B). An impairment of sphere formation was seen in the SK-N-BE(2) *hsa-miR-135b-5p* stable knockdown. *hsa-miR-135b-5p* KD1, KD2 and KD3 were from the same clone. Data represents three independent experiments, presented as the mean ± SEM.

To investigative the possible functional relevance in “stemness” of *hsa-miR-21-5p*, *hsa-miR-181c-5p* and *hsa-miR-135b-5p*, we performed knockdown experiments on Daoy and SK-N-BE(2) cells. Locked Nucleic Acid (LNA™) anti-sense knockdown of *hsa-miR-21-5p* reduced the fraction of ALDH^BR^ cells (surrogate CSC marker) by 50% in Daoy (Figure 3A). We tested the effects of *hsa-miR-181c-5p* and *hsa-miR-135b-5p* inhibition on the ability to grow as neurospheres in Daoy and SK-N-BE(2) respectively. In both cases, the ability to form spheres (read-out for self renewal) was impaired. Results from *hsa-miR-135b* knockdown in SK-N-BE(2) are shown in Figure 3B. Altogether, these observations suggested a possible role of *hsa-miR-21-5p*, *hsa-miR-181c-5p* and *hsa-miR-135b-5p* in regulating self-renewal in Daoy and SK-N-BE(2) CSCs.

## Discussion

In this study we identified a common signature of de-regulated miRNAs associated with CSCs and predicted some candidate pathways affected in CSCs from pediatric tumors. The CSC fraction within the six cell lines was collected using different surrogate assays. Twenty-six differentially expressed miRNAs (DEmiRs) were identified in the CSC fraction of 4 out 6 cell lines. This included miRNAs with known role in CSCs function (such as *hsa-miR21-5p*) but also novel miRNAs (such as *hsa-miR-487b* and *hsa-miR-323-3p*). It was striking to see that 6 of the CSC associated DEmiRs have been reported by other groups as miRNAs implicated in CSC function(s) by regulating core developmental pathways [22]–[28]. To gain insight into the plausible role of the 26 CSC associated DEmiRs, we knocked down 3 of the CSC associated DEmiRs with known functions on CSCs. We demonstrated that *hsa*-*miR-21-5p, hsa-miR-181c-5p* and *hsa-miR-135b-5p* had an effect on the CSC fraction measured using ALDH^BR^ and neurosphere formation as surrogate markers. Our data were similar to studies by other groups that demonstrated an involvement of these DEmiRs in CSC pathways [22]–[28]. These observations suggested that further studies are needed to characterize these CSC associated DEmiRs in pediatric cancers.

We also introduced a systems biology method to predict pathways and functions of these CSC associated DEmiRs. A Bayesian approach BCmicrO [21],[29] was employed as an integrator of multiple target prediction algorithms. A concise mathematical model (Eq. 2) was presented to map miRNAs to pathway regulation or to other functional groups. Enrichment analysis for differentially expressed genes was successfully applied, along with the Fisher’s exact test to determine significant active pathways in our study. Our method provided a unique enrichment analysis that combined the differential expressions of DEmiRs and the target prediction strength to understand the regulation at a pathway or functional level by focusing on the predicted DEmiRs target genes. By using the differential expression level of DEmiRs, we expected further insight into DEmiRs’ regulation and their functions through our method.

Although the functional effect of most miRNAs is still unknown, we utilized the miRNA-targeted genes predicted *in silico* to discover the functions of the DEmiR’s regulation and possible intervention. Our method thus provides a bridge from DEmiRs to pathway and function regulation. The computational analysis of these 26 CSC associated DEmiRs revealed that these DEmiRs might only regulate four annotated pathways: cell proliferation, cell cycle, p53 and TGF-beta/BMP (Tables 1 and 2). It is well known that Notch, Wnt, Hedgehog and PTEN/Akt are core developmental signaling pathways that orchestrate cell proliferation, cell cycle and cell fate *in vivo*. De-regulation in these four main signaling pathways has extensively been demonstrated in CSCs. Our CSC associated DEmiRs are involved in those core developmental signaling pathways, as indicated above. Six of the 26 CSC associated DEmiRs: *hsa*-*miR-19a-5p* [22], *hsa*-*miR-25-5p* [23],[24], *hsa-miR-181c-5p* [25], *hsa-miR-21-5p* reviewed in [26], *hsa*-*miR-135b-5p* [27] and *hsa-miR-148a-5p* [28] have known function(s) in CSCs. In this regard, biologically validated targets of *hsa-miR-21-5p* and *hsa-miR-135b-5p*, as listed in Table 4, have been mapped to PTEN/Akt, Notch and/or Wnt pathways and are essential for CSC self-renewal in different cellular contexts [26],[27]. Our computational analysis mapped the differentially expressed *miR-181c-5p* to Notch signaling pathway (Table 4) and to bone morphogenetic protein (BMP) pathway (Figure 2B) another CSC self-renewal pathway, both known to crosstalk with Wnt (reviewed in [30]). Therefore, we can surmise that in our model systems, *hsa-miR-21-5p*, *hsa-miR-135b-5p* and *hsa-miR-181c-5p* might be regulating cell proliferation and cell cycle likely by affecting PTEN/Akt, Notch and/or Wnt signaling pathways.

**Table 4:** CSC associated DEmiRs and targeted pathways that do not meet following criterion as described in the Results Section: Percentage of miRNA target genes in a gene set is less than 10% or less than 3 genes are regulated by DEmiRs per pathway.

Pathways (database, # of genes)	Target:miR pairs	
Notch Signaling Pathway (KEGG, 47)	*(JAG1:hsa-miR-21-5p)*	
	*(EP300:hsa-miR-132-3p)*	
	*(NOTCH4:hsa-miR-181c-5p)*	
Wnt Signaling Pathway (KEGG, 151)	*(LRP6:hsa-miR-183-5p)*	*(LRP6:hsa-miR-629-3p)*
	*(FZD4:hsa-miR-101-3p)*	*(FZD4:hsa-miR-655)*
	*(FZD6:hsa-miR-101-3p)*	*(FZD6:hsa-miR-655)*
	*(NLK:hsa-miR-181a-5p)*	*(NLK:hsa-miR-181c-5p)*
	*(NFAT5:hsa-miR-29b-3p)*	*(CCND2:hsa-miR-135b-5p)*
	*(EP300:hsa-miR-132-3p)*	
Hedgehog Signaling Pathway (KEGG, 56)	*(CSNK1G1:hsa-miR-494)*	
AKT Pathway (BioCarta, 22)	*(FOXO1:hsa-miR-183-5p)*	

However, the regulation is biologically important in a given pathway.

In summary, we surveyed 6 pediatric cancer cell lines for CSC associated DEmiRs that might regulate CSC functions. Using both target prediction scores and differential miRNAs’ expression, and a novel enrichment algorithm specifically designed for miRNAs expression profiles we predicted pathways that were targeted by these DEmiRs in CSC. Given the importance of developmental pathways such as Notch and Wnt, our findings presented in this study suggested additional targets for prognostic and therapeutic intervention in pediatric cancers.

## Materials and Methods

### Cell Lines

Human pediatric cancer cell lines Daoy (medulloblastoma); SK-N-BE(2) and SH-SY5Y (neuroblastoma), HepG2 (hepatoblastoma), RD-ES (Ewing’s sarcoma), and MG-63 (osteosarcoma) were obtained from American Type Culture Collection (ATCC) and maintained in the ATCC recommended culture medium. All culture media were supplemented with 10% fetal bovine serum (FBS; Atlanta® Biologicals) and 1% antibiotic-antimycotic, amphotericin B, penicillin and streptomycin (Gibco®, Life Technologies™). Hep 293TT (hepatoblastoma) is a new human liver tumor cell line established in our laboratory derived using primary tumor tissues from a 5-year old Caucasian female child [31]. Hep293TT cells were cultured in RPMI 1640 medium containing L-glutamine and 25 mM HEPES (Mediatech) and supplemented with 10% FBS (Atlanta® Biologicals) and 1% antibiotic-antimycotic, amphotericin B, penicillin and streptomycin (Gibco®, Life Technologies™). Cell lines were maintained in a humidified incubator at 37°C with 5% carbon dioxide.

### Neurosphere Formation Assay

An *in vitro* selective culture system referred to as the neurosphere assay was used to enrich Daoy, SK-N-BE(2), SH-SY5Y, RD-ES and MG-63 CSCs. Using a well defined protocol, and in the presence of growth factors including basic fibroblast growth factor (bFGF) and epidermal growth factor (EGF), it is possible to produce a renewal source of stem-like cancer cells, which can be expanded as neurospheres. Cells were trypsinized and resuspended at 2×10^4^ cells/mL then plated onto ultra low attachment plates (Corning) in serum-free maintenance medium for human embryonic stem cells (mTeSR™1, Stem Cell Technologies). After about 2 days, primary spheres were formed with about 100 cells per sphere. To evaluate self-renewal, individual spheres were harvested and disaggregated into single-cell suspensions and serially passaged for two generations.

### Fluorescence Activated Cell Sorting (FACS) and ALDEFLUOR® Assay

Hep293TT and HepG2 stem-like fractions were enriched using FACS to identify cells with high enzymatic activity of Aldehyde Dehydrogenase (ALDH) using the ALDEFLUOR® Assay (Stem Cell Technologies) according to the manufacturer’s instructions. This assay enriched the cell fraction with the high ALDH activity as read-out for the stem-like population. Briefly, BODIPY™ Aminoacetaldehyde (BAAA), a substrate for ALDH, was added, and when excited at 488 nm, the stem-like cells with high ALDH activity were detected and gated by their green fluorescence at 515–545 nm. This purification of stem-like cells was confirmed using diethylaminobenzaldehyde (DEAB), a specific inhibitor of ALDH, as a negative control. Cell sorting was performed with a FACS Aria (FACS Diva v 6.1.2 software; Becton Dickinson). Viable cells were gated using propidium iodide (PI).

### RNA Extraction and High-throughput miRNAs Quantification

Cells were harvested and total RNA was isolation using the mirVana miRNA Isolation Kit (Ambion) according to instructions of the manufacturer. Integrity checks (measured as RNA Integrity Number; RIN) and sample quantitation was performed using the Agilent 2100 Bioanalyzer (Agilent Technologies). miRNA profiling was performed using a high-throughput rapid stem-loop quantitative polymerase chain reaction (qRT-PCR) assay in a high-throughput 384 wells format (MicroRNA Micro Fluid Card Assays’ chemistry; Applied Biosystems) according to the manufacturer’s protocol A total of 768 matured miRNAs and controls found in the human genome, annotated in the miRBase Registry (http://microrna.sanger.ac.uk) were profiled. In the first step, cDNA was reverse transcribed from total RNA samples (2 ng/mL dilution) using two pools of specific stem-loop RT primers. The transcribed cDNAs were loaded onto two sets of microfluidic cards (pool A and B), each pool containing the dried up unique Taqman primers and probes (375 miRNAs and 9 normalization control). qRT-PCR was used to amplify cDNA using specific PreAmp primers and was performed on a ABI Prism 7900HT real-time PCR system in a 384 well format; with pool A and B running separately. Here, the assays were performed in triplicates in two independent experiments. qRT-PCR thermal cycling conditions included 50°C for 2 minutes, 95°C for 5 min, then 40 cycles of 95°C for 15 seconds, 58°C for 30 seconds, 72°C for 30 seconds. The data from the stem-like enriched fractions and their non stem-like counterparts was analyzed using the comparative C_T_ threshold cycle method (2^−ΔΔCT^) method of relative quantification (ΔΔCt = ΔCt sample−ΔCt calibrator; where ΔCt sample = Ct stem-like cells−Ct stem-like cell normalization control, and ΔCt calibrator = Ct non stem-like cells−Ct non stem-like cell normalization control).

### Knockdown of hsa-miR-135b-5p

miRZip-135b anti-miR-135b miRNA construct was purchased from System Biosciences (SBI). Constructs arrived as bacterial streaks. A single colony was picked and grown in LB broth (Fisher Scientific) with 50 µg/mL of ampicillin (Gibco®, Life Technologies™). Plasmids were incubated overnight at 37°C and collected the next day. Plasmids were purified according to the PureLink® Quick Plasmid Miniprep Kit (Life Technologies™) manufacturer’s protocol. Purified plasmid DNA was transfected into SK-N-BE(2) cells. Briefly, SK-N-BE(2) cells were seeded in a 6-well plate at 8×10^5^ cells/well. Cells were grown in antibiotic free growth medium overnight. Cells were transfected with 4 µg of DNA using Lipofectamine 2000 (Life Technologies™) according to the manufacturer’s protocol. Briefly, DNA and Lipofectamine were incubated in separate tubes containing 250 µL of OptiMEM (Gibco®, Life Technologies™). Plasmid DNA and Lipofectamine were then combined and incubated for 20 min. Cells were washed with OptiMEM and fresh OptiMEM was added to each well. The plasmid DNA and Lipofectamine mix was then added to each designated well and incubated for 24 hours. After 24 hours, the GFP expression was measured to determine transfection efficiency. To generate a stable cell line, cells were passaged and grown in growth medium containing 1 µg/mL of puromycin (Gibco®, Life Technologies™).

### Knockdown of *hsa-miR-21-5p* and *hsa-miR-181c-5p* with Anti-sense LNA™ Oligonucleotides

Daoy cells in exponential phase of growth were plated in 6-well plates (TPP, Techno Plastic Products) at 8×10^4^ cells/well and cultured in antibiotic free media for ∼24hours, followed by transfection of miRCURY LNA™ knockdown probes for *hsa-miR21-5p* and *hsa-miR181-5pc* (Exiqon Inc.), using oligofectamine and OPTIMEM® serum-free medium (Life Technologies™), according to the manufacturer’s protocol. About 6 hours after transfection, media with serum was added, then 24 hours after transfection, media was changed to its regular growth medium (Minimum Essential Medium Eagles (Mediatech), 10% FBS (Atlanta® Biologicals) and 1% antibiotic-antimycotic; amphotericin B, penicillin and streptomycin (Gibco®, Life Technologies™). No probe (mock) was used as negative control.

### Statistical Data Analysis for miRNA Profiling

#### MicroRNA expression quality control

MicroRNA expression data generated with microfluidic cards assays were first examined by scatter plots comparing all samples (Figure S1 in File S1) where miRNA expression levels (ΔCt) of stem-like enriched fractions and their non stem-like counterparts were plotted. The results should exhibit some level of concordance (we choose correlation coefficient to be greater than 0.75), otherwise, a replication will be called to confirm the outcome or replace the failed assay.

#### Differentially expressed miRNAs (DEmiRs) selection

From 6 stem-like expression profiles, DEmiRs were selected by requiring their expression levels were at least 2-fold up- or down-regulated comparing to their non stem-like counterparts in at least 1 cell-line (miRs with no expression readouts were considered as non-differentially expressed miRs), and no more than 2 cell-lines without expression readouts. To examine miRNAs that show consistent differential expression among 6 CSCs, we chose at least DEmiRs in at least 4 out 6 CSCs to allow some level of fault-tolerance yet consistence as the candidate CSC signature miRNAs. Assuming each miR has the probability of 0.2 (20% DEmiRs over total miRNAs in the genome) to be differentially expressed, a miRNA is differentially expressed in at least 4 out of 6 cell-lines by chance is 0.017 (binomial distribution), or we expect about 1 in 58 differentially miRNAs selected due to random chance.

#### Hierarchical clustering of DEmiRs

Hierarchical clustering method was employed to observe concerted miRNA expression pattern of CSCs. We first selected all probes with no more than 2 blank measurements among 6 cell-lines (total of 380 probes were selected). For the remaining probes without expression measurement, imputation of *k*-nearest neighbor method (*k* = 3, distance measurement is set to use correlation coefficient) was applied to impute the expression values (MATLAB Bioinformatics Toolbox, Mathworks). Hierarchical clustering algorithm was then applied to log2-transformed miRNA expression value by using Spearman correlation coefficient as distance measure, and average linkage for dendrogram. Samples with similar miRNA expressions are expected to group together. Annotation of miRNAs was obtained from miRBase (http://mirbase.org, Release 19, August 2012) via sequence search.

### The Prediction of MicroRNA Target Genes

miRNA target genes were collected from two sources: 1) experimentally validated targets, and 2) computational predicted targets. We chose all experimentally validated targets, complemented with a set of predicted targets compiled from multiple prediction algorithms with an innovative Bayesian-based algorithm. Briefly, 1) validated miRNA target genes were generated from the MiRecords database, which provides curated experimentally validated miRNA targets from published literature [32] and 2) a novel Bayesian based miRNA target prediction algorithm BCmicrO [21],[29], where the distributions of prediction results for each algorithm were explicitly modeled based on a training dataset composed of carefully constructed positive and negative miRNA-target pairs. Briefly, prediction scores of 6 algorithms (TargetScan, miRanda, PicTar, mirTarget, PITA and Diana-microT) were examined. For example, TargetScan predicts miRNA’s potential binding sites in the mRNA’s 3′ UTR, where a context score is calculated for each site and the total context score for a 3′UTR is computed to represent the confidence of the corresponding mRNA to be a target. Let *x*_1_, *x*_2_, …, *x*_6_ denote the scores of a miRNA-mRNA pair by TargetScan, miRanda, PicTar, mirTarget, PITA and Diana-microT, respectively, and let y be an indicator variable such that *y* = 1, if the mRNA is a real miRNA target, and *y* = 0, otherwise. BCmicrO thus calculates the probability *p*(*y* = 1| *x*_1_, *x*_2_, …, *x*_6_), the posterior probability of the mRNA to be the miRNA’s target given the 6 algorithms’ prediction scores, which can be expressed as
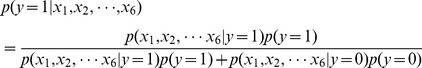
(1)where *p*(*y* = 1) is the prior probability of an mRNA being a target and and *p*(*x*_1_, *x*_2_, …, *x*_6_|*y*) is the likelihood function. It is clear that *p*(*x*_1_, *x*_2_, …, *x*_6_|*y* = 1) can be estimated from the validated target set defined by miRecords. BCmicrO was trained by 929 positive and 23,319 negative miRNA-target pairs derived from 20 GEO data sets with microRNA over-expression and from work of Karginov *et al*. [33]. Particularly, the gamma distributions were used to model the likelihood function of each algorithm’s prediction scores given validated targets (*y* = 1) or negative targets (*y* = 0). We chose probability >0.6 to be our miRNA-mRNA pairs before submitting for pathway and function enrichment.

### Gene Sets of KEGG, Biocarta Pathway and Gene Ontology

The gene sets of KEGG and Biocarta pathways were downloaded from the Molecular Signature Database MsigDB 3.0 (http://www.broadinstitute.org/gsea/msigdb/index.jsp) [9]. Included in the MsigDB, 186 and 217 gene sets are designated to KEGG and BioCarta pathways, respectively. For Biological Processes in Gene Ontology, we downloaded from the BioMart website of Ensembl (http://useast.ensembl.org/info/data/biomart.html) with version Homo Sapiens 65 [34]. A total of 7,654 GO terms was downloaded and finally 1,091 gene sets associated to GO terms remained after excluding the gene sets containing fewer than 15 genes.

### Enrichment Analysis of miRNA Regulated Gene Set

The effects of the DEmiRs on the miRNA target genes were determined by using the following equation,
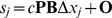
(2)where Δ**x** *_j_* is a vector of the differential expression levels (i.e., log-ratio) of miRNAs of the *j* ^th^ cell line; **B** is the target prediction scoring matrix (discussed in previous section), or **B** = (*b_ik_*)*_M_*_x*K*_, where *M* is the number of genes and *K* the number of miRNAs, and matrix **P** = {*p_l,i_*}*_L_*_x*M*_ be an indicator matrix of gene sets, where *p_l,i_* = 1 when *i* ^th^ gene is in the *l* ^th^ gene set, otherwise 0. Then the miRNA expression values can be transferred as the strength of gene regulation exerting on each pathway for each cell-line, or **s** *_j_* = {*s*_1,*j*_, …, *s_L,j_*}, and *s_i,j_* is the aggregated regulation in *i* ^th^ gene set derived from all differential expressed miRNAs of the *j* ^th^ cell-line. The enrichment score (ES) is defined as,
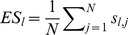
(3)where ES>0 represents the enriched function of a given pathway, ES<0 represents the depletion of a pathway function, and ES = 0 indicates no enrichment. In our analysis, to evaluate the significance, we chose ES>1.5 to represent the enrichment of a pathway or gene-set function and also used Fisher’s exact test to evaluate the significance of each enriched pathway by considering the total number of genes regulated in the genome and in a pathway, as well as the number of genes in the genome and in a pathway. To select a enriched pathway or gene set, it has to satisfy three criteria: 1) Percentage of miRNA target genes in a gene set exceeds 10% and at least 3 genes are regulated by DEmiRs, 2) Enrichment score ES>1.5; and 3) P value of Fisher’s exact test is less than 0.01. We chose not to perform random permutation here for the ES due to the relative few cell-lines under consideration and much fewer miRNAs than number of genes commonly used in gene expression data analysis.

## Supporting Information

File S1
**Supplemental data. Figure S1,** Scatter plots of raw ΔCt of 6 cell lines between test (enriched CSC miRNAs) and reference (non-enriched counterpart) samples before normalization. **Figure S2,** Heatmap of 865 putative microRNA target genes computed by 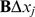. 630 of the putative microRNA target genes were up-regulated and 235 were down regulated. The values of 865 target genes were computed by 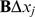 (see Eq. 2 in Methods Section for details). **Figure S3,** The CSC associated DEmiRs regulated Biocarta pathways. (A–D) CELLCYCLE, G1, P53, and RACCYCD pathways, (E–H) CTCF, TOB1, ALK, and PTEN pathways. **Table S1,** Cell cycle and cell proliferation related genes in the cancers. (√ indicates the gene is in the gene set named in the column header).(PDF)
