# Supplementary material for: De-Regulated MicroRNAs in Pediatric Cancer Stem Cells Target Pathways Involved in Cell Proliferation, Cell Cycle and Development
Source: PLoS One. 2013 Apr 17;8(4):e61622. doi: 10.1371/journal.pone.0061622 (PMC3629228; doi:10.1371/journal.pone.0061622)
Supplement: File S1 — Supplemental data. Figure S1, Scatter plots of raw ΔCt of 6 cell lines between test (enriched CSC miRNAs) and reference (non-enriched counterpart) samples before normalization. Figure S2, Heatmap of 865 putative microRNA target genes computed by . 630 of the putative microRNA target genes were up-regulated and 235 were down regulated. The values of 865 target genes were computed by (see Eq. 2 in Methods Section for details). Figure S3, The CSC associated DEmiRs regulated Biocarta pathways. (A–D) CELLCYCLE, G1, P53, and RACCYCD pathways, (E–H) CTCF, TOB1, ALK, and PTEN pathways. Table S1, Cell cycle and cell proliferation related genes in the cancers. (√ indicates the gene is in the gene set named in the column header). (PDF) [file pone.0061622.s001.pdf]

Supplemental Data

De-regulated microRNAs in pediatric cancer stem cells target pathways involved in cell proliferation, cell cycle and development

Patricia C. Sanchez Diaz, Tzu-Hung Hsiao, Judy C. Chang, Dong Yue, Mimi C. Tan, Harry Hung-I Chen, Gail E. Tomlinson, Yufei Huang, Yidong Chen, and Jaclyn Y. Hung

Table S1. Cell cycle and cell proliferation related genes in the cancers. (√ indicates the gene is in the gene set named in the column header).

	CHRONIC MYELOID LEUKEMIA	GLIOMA	MELANOMA	PANCREATIC CANCER	PROSTATE CANCER	SMALL CELL LUNG CANCER
**CDKN1A**	√	√	√	√	√	
**CDKN1B**	√			√	√	√
**E2F1**	√		√	√	√	√
**RB1**	√	√	√	√	√	√
**PTEN**		√	√		√	√

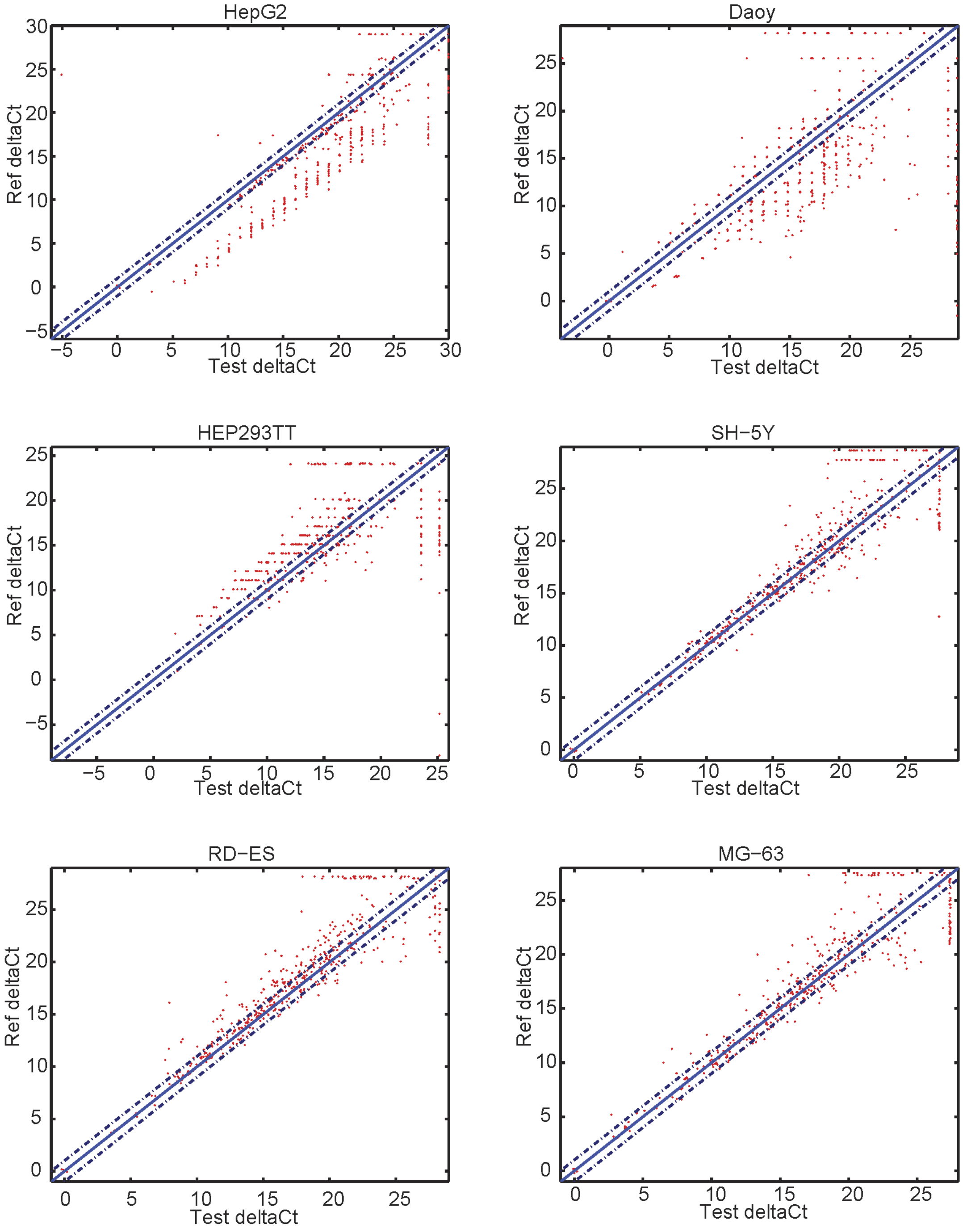

Figure S1. The scatter plots of raw ΔCt of 6 cell lines between test (enriched CSC miRNAs) and reference (non-enriched counterpart) samples before normalization.

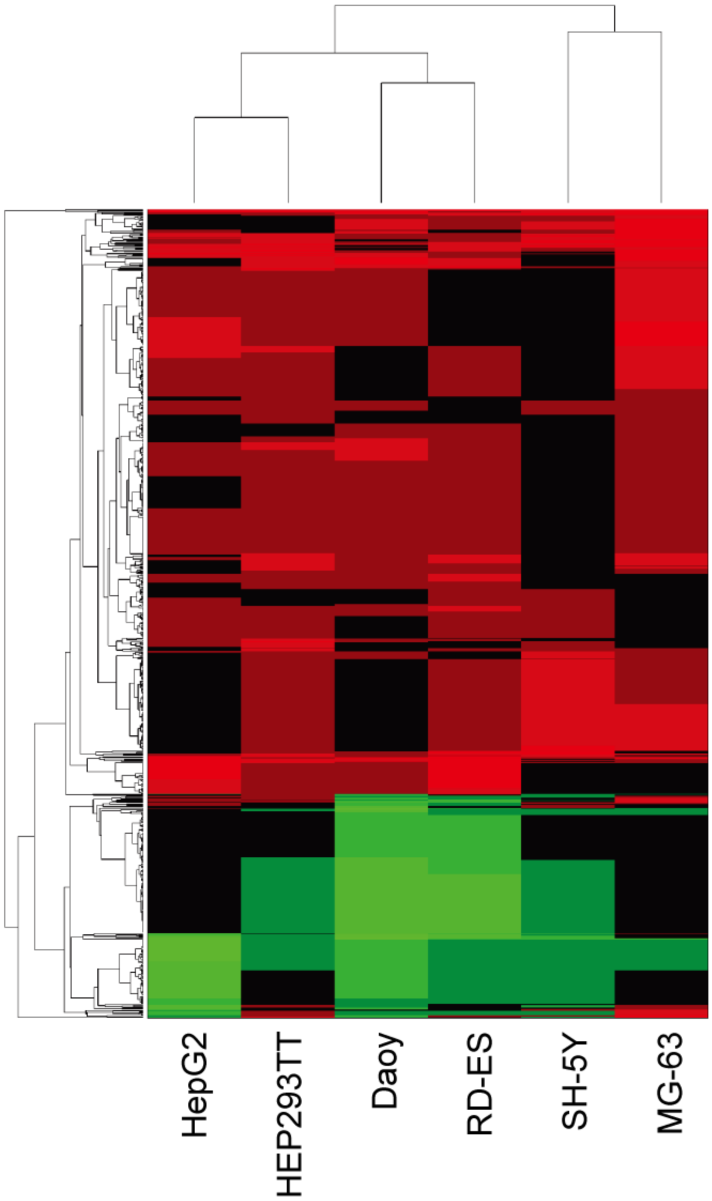

Figure S2. The heatmap of 865 putative microRNA target genes. 630 of 865 putative microRNA target genes were up-regulated and 235 were down regulated. The 863 target genes' value were computed by $\mathbf{B}\Delta x_j$ (see Eq. 2, Materials and Methods Section for details).

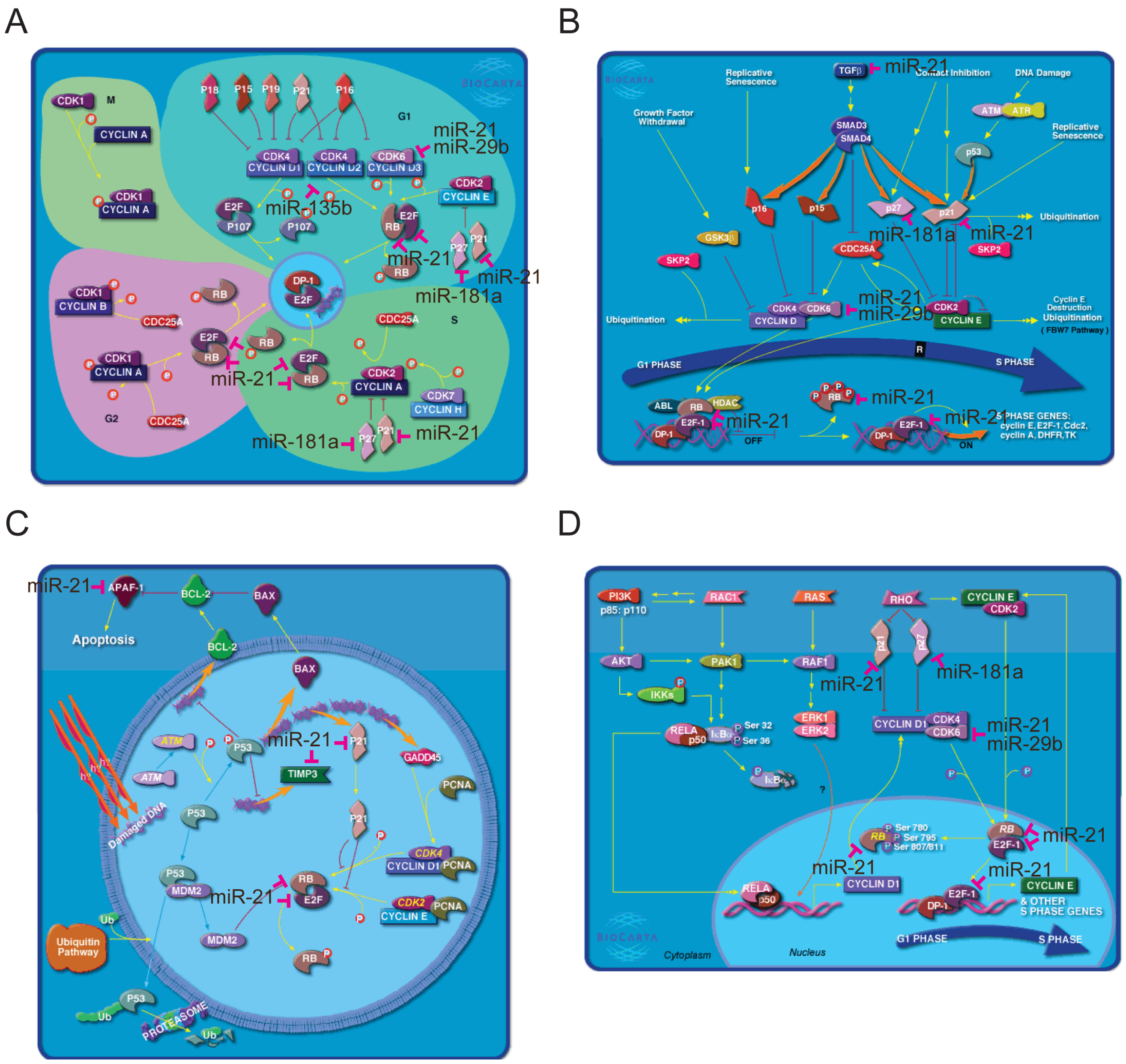

Figure S3-1. The CSC associated Biocarta pathways regulated by miRNAs identified in Figure 1B. A) CELLCYCLE, 2) G1, 3) P53, and 4) RACCYCD pathway.

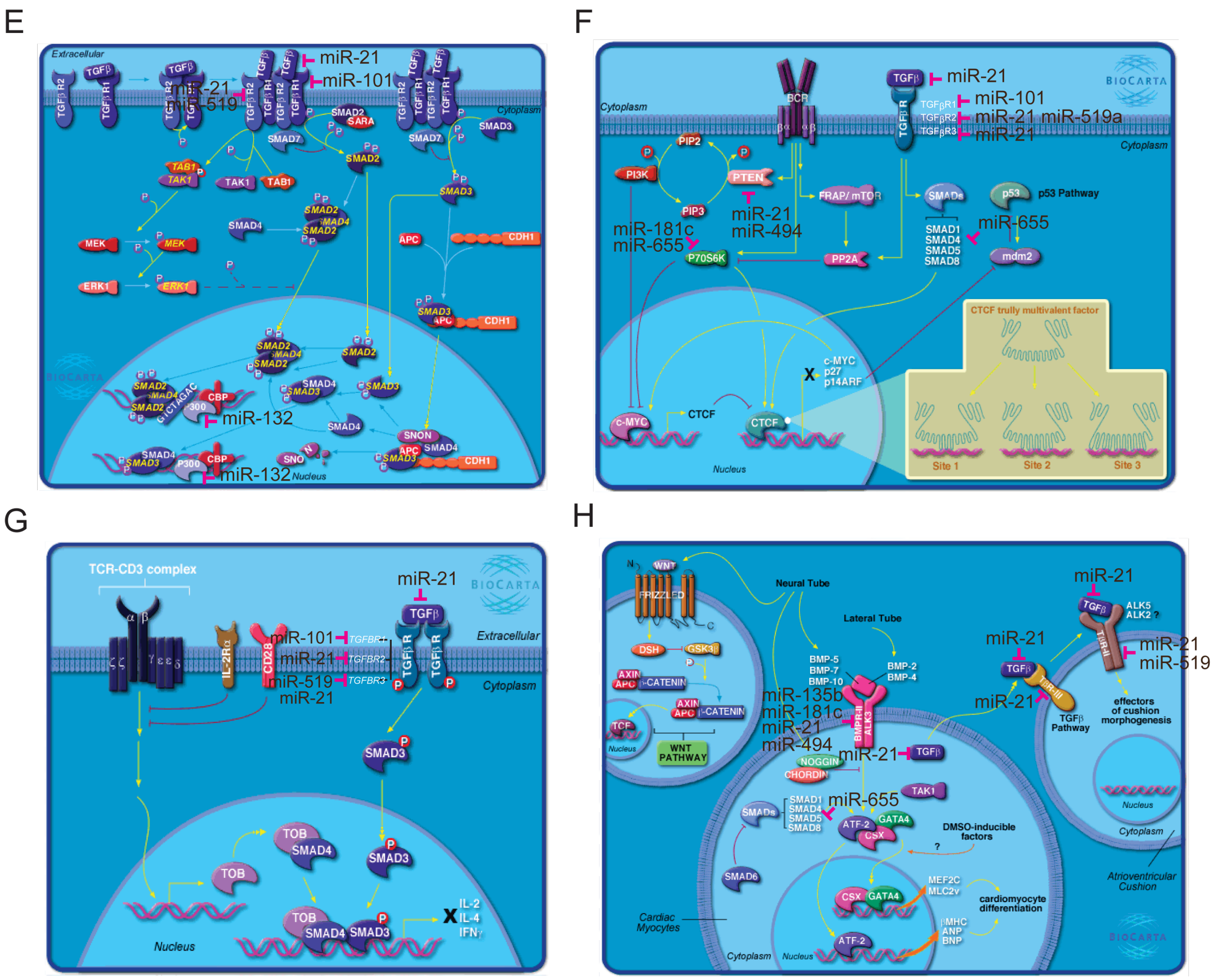

Figure S3-2. The CSC associated Biocarta pathways regulated by miRNAs identified in Figure 1B. E) CTCF, F) TOB1, G) ALK, and H) PTEN pathway.
